# Psychological impact on dental students and professionals in a Lima population during COVID-19s wave: a study with predictive models

**DOI:** 10.1038/s41598-022-18899-x

**Published:** 2022-08-30

**Authors:** Mariana Morales-Montoya, Nancy Córdova-Limaylla, Gissela Briceño-Vergel, Marysela Ladera-Castañeda, Goretty Garcia-Luna, Hernán Cachay-Criado, Luis Cervantes-Ganoza, César F. Cayo-Rojas

**Affiliations:** 1 School of Stomatology, Universidad Privada San Juan Bautista, Av. Jose Antonio Lavalle Avenue s/n (Ex Hacienda Villa); Chorrillos, Lima, Peru; 2 Postgraduate School, Grupo de Investigación Salud Y Bienestar Global”, Universidad Nacional Federico Villarreal, Lima, Peru; 3 Faculty of Stomatology, Universidad Inca Garcilaso de la Vega, Lima, Peru

**Keywords:** Psychology, Risk factors

## Abstract

Peru was the country with the highest COVID-19 case fatality rate worldwide during second wave of infection, with dentists and pre-professional students being susceptible to infection due to clinical procedures they perform. This situation could have generated some kind of psychological disorder within this group. Therefore, the present study aimed to assess how COVID-19 pandemic affected this population group during second wave, in relation to depression, anxiety and stress. This observational and cross-sectional study in 368 Peruvian dentists (186 students and 182 professionals), was carried out during August to November 2021. The DASS-21 Scale was used to diagnose depression, anxiety and stress. For the statistical analysis, Pearson's chi-square test was used, in addition to a logit model using odds ratio (OR) to evaluate depression, anxiety and stress with the following factors: gender, age group, marital status, monthly family income, children, academic level, history of COVID-19, COVID-19 symptomatology, close relative with COVID-19, living with vulnerable people and work dedication. In addition, predictive models were constructed considering all possible significant causes. A significance level of p < 0.05 was considered. Dental students and professionals presented significant differences in levels of depression, anxiety and stress (p < 0.001, p = 0.022, p = 0.001; respectively). Male students were 56% less likely to develop stress (OR 0.44; CI 0.22–0.85) compared to females; while those unmarried were 81% less likely to develop stress (OR 0.19; CI 0.04–0.85). Likewise, those with children were 83% less likely to develop stress (OR 0.17; CI 0.06–0.52) and 65% less likely to develop depression (OR 0.35; CI 0.15–0.80). In addition, COVID-19 asymptomatics were 60% less likely to develop depression (OR 0.40; CI 0.17–0.92). However, having relatives with COVID-19 caused almost three times the probability of developing depression (OR 2.96; CI 1.29–6.79) and twice the probability of developing stress (OR 2.49; CI 1.07–5.78). As for dental professionals, it was noticed that those unmarried had almost three times the probability of developing stress (OR 2.93; CI 1.38–6.23); while those who only worked had twice the probability of developing stress (OR 2.37; CI 1.17–4.78). Dental students had a higher prevalence of depression, anxiety and stress. In addition, having children and being asymptomatic were protective predictors for depression, while being male, unmarried and having children were protective predictors for stress. However, having a relative with COVID-19 was a risk predictor for depression and stress. In professionals, only working and being unmarried were risk predictors for stress.

## Introduction

In the last two years, due to COVID-19, a worldwide crisis occurred in all social classes because no country was prepared to face the repercussions of pandemic such as increased unemployment rate, change of habits, migration to virtual labor and education, as well as loss of relatives^1–4^. In this context, medical and dental care was limited, forcing many dentists to close their dental offices and clinics, generating job uncertainty and a possible psychological impact^5,6^.

In May 2020, Peru began the process of opening its labor market and in its second stage, the opening of dental care was allowed under the condition of complying with all the protocols indicated by the Ministry of Health. This implied an additional expense for the professionals due to the reduction of suppliers and increase in cost of personal protective equipment, causing even greater concern^4,7^. On the other hand, university students face personal, social, emotional and academic situations that make them more vulnerable to psychological disorders^8^. Also, in the current context dental students had to migrate to virtual education, which represented an important change in the way of learning as they were not used to it, affecting the acquisition of motor skills that are provided by clinical training^9^. In addition, many were affected by the uncertainty of being able to continue with their professional career due to the new economic and social situation, which could lead to some type of behavioral disorder^10–14^. Likewise, given the characteristics of dental care that involves emissions of biocontaminated aerosols, it is important that students are prepared in the cognitive and practical aspects of infection control^15^, because if they do not have sufficient knowledge, it could generate some behavioral disorder by thinking that the return to clinical care will expose them to possible COVID-19 infections.

In Peru, it was thought that the COVID-19 infection curve would decrease (December 2020) and flatten out with the arrival of vaccines, returning to normal by 2021. However, the population was suddenly confronted with a second wave of infections with a higher case fatality rate worldwide, peaking at the end of April and beginning of May 2021 and remaining until June, decreasing only in November 2021^16^. Because of this, it is important to evaluate the psychological state of frontline health personnel in the face of a potentially infected patient, such as dental professionals and students who had already begun their pre-professional training, since it was reported that dentists developed anxiety, stress and depression during the first wave of infection, as they felt more prone to become ill with COVID-19 due to frequent exposure to contaminated salivary aerosols during health care procedures^17–19^.

Anxiety has been defined as a physiological response of the organism to counteract or override an imminent threat or danger^20,21^; while stress has been defined as the set of neuroendocrine, immunological, emotional and behavioral responses to situations that demand greater adaptation than usual^21,22^. Depression has been defined as a state of emotional pain, unhappiness or sadness that manifests as a reaction to an unpleasant event or situation^22,23^. In addition, it has been reported that several sociodemographic factors are associated with some psychological disorders in the context of pandemic and confinement, such as age, gender, marital status, occupation (student or professional), place of residence, number of children, economic difficulties, among others^11,14,24–26^.

There are several instruments to reliably measure psychological disorders subjectively. The DASS-21 scale has been widely used internationally by numerous researchers, since it measures anxiety, stress and depression, with an acceptable psychometric performance^27–29^.

Therefore, aim of the present study was to evaluate the factors associated with the levels of anxiety, stress and depression in dental professionals and students in a Lima population during the second wave of COVID-19 infection. This manuscript was written according to the STrengthening the Reporting of OBservational studies in Epidemiology (STROBE) guidelines for observational studies^30^.

## Methods

### Type of study and delimitation

This analytical, prospective, observational and cross-sectional study was carried out on dental students and professionals at two universities in the Peruvian capital between August and November 2021.

### Population and selection of participants

The total population consisted of 532 participants, including dental students and professionals, of whom 118 and 220 were students from the Universidad Privada San Juan Bautista (UPSJB) and the Universidad Nacional Federico Villarreal (UNFV), respectively; while the professionals were 81 from the UPSJB and 113 from the UNFV. The minimum sample size was 168 participants per group, this was calculated with a formula for comparison of proportions for independent groups considering a significance level α = 0.05 and a statistical power 1 − β = 0.80, and a prevalence of students P_1_ = 0.77 and professionals P_2_ = 0.63. These proportions were obtained from a pilot study with 60 participants (30 students and 30 professionals).

The sample selection technique was systematic randomization for both groups and taking into consideration the eligibility criteria, 368 participants were included as the final sample (n = 368; 186 students and 182 professionals).

#### Inclusion criteria

1. Students of both sexes over 18 years of age.
2. Dental students in their 5th and 6th year of academic training.
3. Students enrolled in the second semester of year 2021.
4. Graduate dental professionals teaching or studying at UPSJB or UNFV.
5. Students and dental professionals who accepted the virtual informed consent.
6. Peruvian students and dental professionals residing in Lima.

#### Exclusion criteria

1. Students with truncated studies.
2. Students and professionals who did not complete the questionnaire.

### Associated factors

Associated factors considered in the study, in relation to development of depression, anxiety and stress were: gender, age group, marital status, monthly family income, children, academic level, history of COVID-19, COVID-19 symptomatology, close relative with COVID-19, living with vulnerable people and occupation.

### Application of the instrument

The instrument used was the DASS-21 scale, which consists of 21 items distributed in three dimensions: depression, anxiety and stress. Each dimension was composed of 7 questions randomly distributed in the questionnaire. In addition, each item had four ordinal (Likert-type) response alternatives: "Never" (0 points), "Sometimes" (1 point), "Frequently" (2 points) and "Almost always" (3 points). Scores obtained from the students and dental professionals in each dimension were added together, which made it possible to diagnose depression, anxiety and stress. Finally, those who scored 5 to 21 points were diagnosed with depression, while those who scored 4 to 21 points were diagnosed with anxiety and finally those who scored 8 to 21 points were diagnosed with stress^31,32^. The DASS-21 subscales were scored as follows: normal (0–4 points), mild (5–6 points), moderate (7–10 points), severe (11–13 points), and extremely severe (14 + points) for depression; normal (0–3 points), mild (4–5 points), moderate (6–7 points), severe (8–9 points), and extremely severe (10 + points) for anxiety; and normal (0–7 points), mild (8–9 points), moderate (10–12 points), severe (13–16 points), and extremely severe (17 + points) for stress^32^.

To evaluate reliability of the instrument, Cronbach's alpha was applied and a significantly acceptable value was obtained: 0.88; (95% CI 0.79–0.97). In addition, the scale was taken at two different times within 7 days to evaluate the concordance analysis of the responses, altering the order of the questions to avoid memory bias (test–retest). The concordance according to Cohen's Kappa index was very good (k = 0.87; 95% CI 0.75–0.98).

### Procedure

The scales developed in Google Classroom® were distributed in a self-administered manner to each student and dental professional, sending the link to their e-mails or through the social networks with WhatsApp®, Twitter® and Facebook®. They were asked to initial their first name, last name and age (for example: CCR40), so that any repetitions could be eliminated. The survey form was also edited to allow only one mailing to the associated e-mail address. Informed consent to participate in the study was given at the beginning of the scale, followed by the indications for its development. However, everyone was free to refuse the evaluation if they did not wish to complete it during the course of the study. Only the researchers had access to the data and no personal details such as telephone number, full name and address were required. Only one submission was considered for each student and dental professional. In addition, after the entire investigation was completed, the results were sent to those who requested them to the principal investigator via email.

### Data analysis

Data analysis was performed with the Statistical Package for the Social Sciences (SPSS) version 24.0. Descriptive statistics were used to obtain the percentages of categorical variables. Pearson's chi-square test with Yates correction was used for bivariate analysis because it determines whether the distribution of the observed response occurs randomly or is significantly associated with a demographic variable. Mann Whitney U test was used to compare the levels of depression, anxiety and stress. Risk factors were examined by logistic regression model (logit model) using odds ratio (OR), assessing statistical assumptions such as the observations were independent, no multicollinearity, and sample size was sufficient according to the number of explanatory variables, in addition to assessing the goodness of fit in the model. All analyses were performed, considering a significance level of 5% (p < 0.05).

### Bioethical considerations

All participants gave informed consent. In addition, the present research respected the bioethical principles for medical research in human beings of the Declaration of Helsinki^33^ elated to confidentiality, freedom, respect, and nonmaleficence; and, it was approved by the Ethics Committee of the Universidad Privada San Juan Bautista. (No. 937-2021-CIEI-UPSJB).

### Ethic approval and consent to participate

The present study respected the bioethical principles for medical research on human beings of the Declaration of Helsinki ^33^, related to confidentiality, freedom, respect and non-maleficence. It was also approved by the Institutional Research Ethics Committee of the Universidad Privada San Juan Bautista with resolution No. 937–2021-CIEI-UPSJB dated Aug 27, 2021. All participants understood and signed an informed consent.

## Results

Of the 368 participating dentists, the mean age of the respondents was 25.3 ± 6.7 years and 38.4 ± 10.4 years for the 186 students and 182 professionals, respectively. The predominant gender was female (62.9%) for students and male (55.5%) for professionals. The majority of students (77.4%) were under 30 years of age, while the majority of professionals (73.1%) were 30 years of age or older. The majority of students (93.0%) and professionals (54.9%) were not married. Regarding family income, 90.9% and 61.0% of the students and professionals, respectively, earned less than US$1125 per month. Of the students, 80.6% reported having no children and 52.2% of the professionals reported having at least one child. Additionally, it could be seen that the majority of students (72.0%) study and work at the same time, while the majority of professionals (57.7%) only work, 47.8% of them holding a Master's degree (Table 1).

**Table 1:** Characterization of sociodemographic variables in dental students and professionals in a Lima population during the second wave of COVID-19.

Variable	Category	Student		Professional		Total	
		f	%	f	%	f	%
Gender	Male	69	37.1	101	55.5	170	46.2
	Female	117	62.9	81	44.5	198	53.8
Age group	< 30 years	144	77.4	49	26.9	193	52.4
	≥ 30 years	42	22.6	133	73.1	175	47.6
Marital status	Unmarried	173	93.0	100	54.9	273	74.2
	Married	13	7.0	82	45.1	95	25.8
Monthly family income	< $1125	169	90.9	111	61.0	280	76.1
	≥ $1125	17	9.1	71	39.0	88	23.9
Children	Yes	36	19.4	95	52.2	131	35.6
	No	150	80.6	87	47.8	237	64.4
Academic level	Bachelor			69	37.9	69	37.9
	Magister			87	47.8	87	47.8
	Doctor			26	14.3	26	14.3
History of COVID-19	Yes	75	40.3	52	28.6	127	34.5
	No	111	59.7	130	71.4	241	65.5
Symptoms of COVID-19	Asymptomatic	35	18.8	21	11.5	56	15.2
	Symptomatic	46	24.7	35	19.2	81	22.0
	Does not refer	105	56.5	126	69.2	231	62.8
Close relative with COVID-19	Yes	148	79.6	127	69.8	275	74.7
	No	38	20.4	55	30.2	93	25.3
Living with vulnerable people	Yes	122	65.6	111	61.0	233	63.3
	No	64	34.4	71	39.0	135	36.7
Occupation	Study	52	28.0			52	14.1
	Work			105	57.7	105	28.5
	Study and work	134	72.0	77	42.3	211	57.3
Age	Mean ± SD	25.3 ± 6.7		38.4 ± 10.4		31.8 ± 10.9	

*f* absolute frequency, *SD* standard deviation.

On the other hand, the majority of students (59.7%) and dentists (71.4%) reported that they had not been ill with COVID-19 since the pandemic began. In that sense, the majority of students (56.5%) and professionals (69.2%) did not report having felt any symptoms of COVID-19 during the pandemic. However, 79.6% of students and 69.8% of professionals report having had at least one close relative with COVID-19. Finally, the majority of students (65.6%) and professionals (61.0%) reported living with people vulnerable to COVID-19. (Table 1).

**Figure 1:**
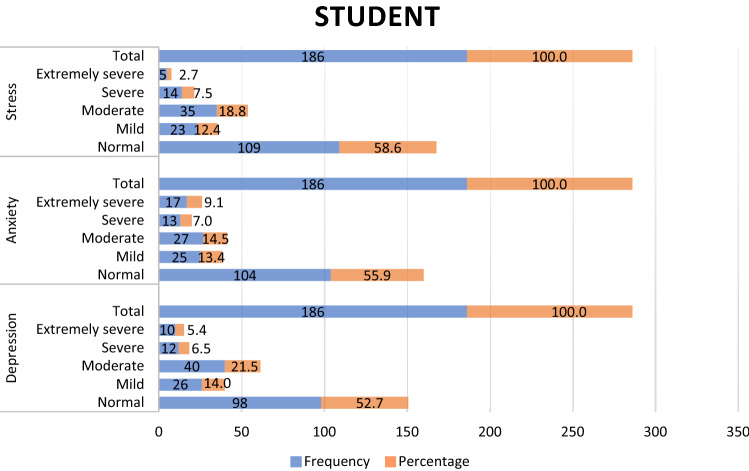
Distribution of dental students according to their levels of Depression, Anxiety and Stress.

**Figure 2:**
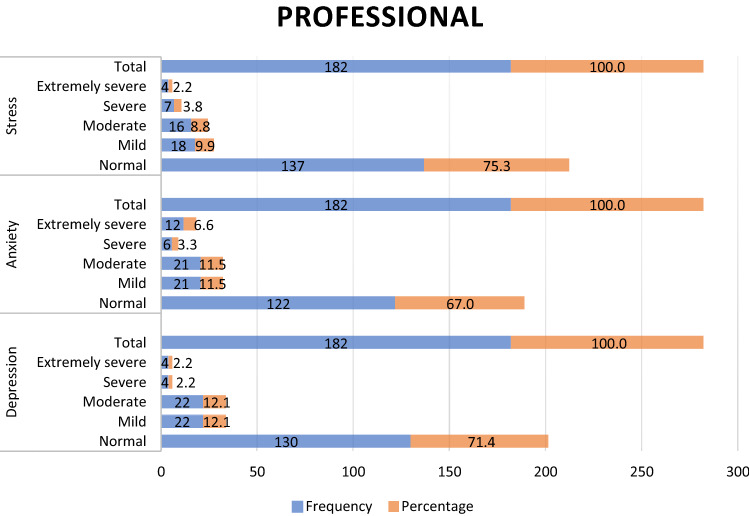
Distribution of dental professionals according to their levels of Depression, Anxiety and Stress.

Of the 186 dental students, 41.4%, 44.1% and 47.3% presented stress, anxiety and depression respectively, with the moderate level being the most prevalent in all of them (Fig. 1). On the other hand, of the 182 dental professionals, 24.7%, 33.0% and 28.6% presented stress, anxiety and depression respectively, with the mild and moderate levels (Fig. 2) being the most prevalent. Therefore, when comparing the levels of depression, anxiety and stress among students and dental professionals, significant differences were observed (p < 0.001, p = 0.022, p = 0.001, respectively) (Table 2).

**Table 2:** Comparison of Depression, Anxiety and Stress levels among dental students and professionals in a Lima population during the second wave of COVID-19.

Variable	Group	f	Nivel					*p
			Normal	Mild	Moderate	Severe	Extremely severe	
Depression	Student	186	98	26	40	12	10	0.000
	Professional	182	130	22	22	4	4	
Anxiety	Student	186	104	25	27	13	17	0.022
	Professional	182	122	21	21	6	12	
Stress	Student	186	109	23	35	14	5	0.001
	Professional	182	137	18	16	7	4	

*f* absolute frequency, Based on Whitney's Mann's U; p < 0.05 (significant differences).

Regarding dental students, depression was significantly associated with having children (p = 0.009), COVID-19 symptomatology (p = 0.020) and having had close relatives with COVID-19 (p = 0.011). In addition, anxiety was significantly associated with gender (p = 0.049), history of COVID-19 (p = 0.007), COVID-19 symptomatology (p = 0.001) and having had a close relative with COVID-19 (p = 0.035). Finally, stress was significantly associated with gender (p = 0.008), having children (p = 0.009) and having had a relative with COVID-19 (p = 0.013) (Table 3).

**Table 3:** Depression, Anxiety and Stress associated with sociodemographic factors of dental students in a Lima population during the second wave of COVID-19.

Variable	Category	Depression			Anxiety			Stress		
		Yes	No	*p	Yes	No	*p	Yes	No	*p
		f (%)	f (%)		f (%)	f (%)		f (%)	f (%)	
Gender	Male	27 (14.5)	42 (22.6)	0.860	24 (12.9)	45 (24.2)	0.049	20 (10.8)	49 (26.3)	0.008
	Female	61 (32.8)	56 (30.1)		58 (31.2)	59 (31.7)		57 (30.6)	60 (32.3)	
Age group	< 30 years	71 (38.2)	73 (39.2)	0.313	65 (34.9)	79 (42.5)	0.592	61 (32.8)	83 (44.6)	0.621
	≥ 30 years	17 (9.1)	25 (13.4)		17 (9.1)	25 (13.4)		16 (8.6)	26 (14.0)	
Marital status	Unmarried	81 (43.5)	92 (49.5)	0.625	75 (40.3)	98 (52.7)	0.462	70 (37.6)	103 (55.4)	0.345
	Married	7 (3.8)	6 (3.2)		7 (3.8)	6 (3.2)		7 (3.8)	6 (3.2)	
Monthly family income	< $1125	82 (44.1)	87 (46.8)	0.298	75 (40.3)	94 (50.5)	0.800	71 (38.2)	98 (52.7)	0.592
	≥ $1125	6 (3.2)	11 (5.9)		7 (3.8)	10 (5.4)		6 (3.2)	11 (5.9)	
Children	Yes	10 (5.4)	26 (14.0)	0.009	15 (8.1)	21 (11.3)	0.745	8 (4.3)	28 (15.1)	0.009
	No	78 (41.9)	72 (38.7)		67 (36.0)	83 (44.6)		69 (37.1)	81 (43.5)	
History of COVID-19	Yes	39 (21.0)	36 (19.4)	0.292	42 (22.6)	33 (17.7)	0.007	32 (17.2)	43 (23.1)	0.773
	No	49 (26.3)	62 (33.3)		40 (21.5)	71 (38.2)		45 (24.2)	66 (35.5)	
Symptoms of COVID-19	Asymptomatic	24 (12.9)	11 (5.9)	0.020	25 (13.4)	10 (5.4)	0.001	16 (8.6)	19 (10.2)	0.829
	Symptomatic	19 (10.2)	27 (14.5)		20 (10.8)	26 (14.0)		18 (9.7)	28 (15.1)	
	Does not refer	45 (24.2)	60 (32.3)		37 (19.9)	68 (36.6)		43 (23.1)	62 (33.3)	
Close relative with COVID-19	Yes	77 (41.4)	71 (38.2)	0.011	71 (38.2)	77 (41.4)	0.035	68 (36.6)	80 (43.0)	0.013
	No	11 (5.9)	27 (14.5)		11 (5.9)	27 (14.5)		9 (4.8)	29 (15.6)	
Living with vulnerable people	Yes	62 (33.3)	60 (32.3)	0.186	54 (29.0)	68 (36.6)	0.947	56 (30.1)	66 (35.5)	0.085
	No	26 (14.0)	38 (20.4)		28 (15.1)	36 (19.4)		21 (11.3)	43 (23.1)	
Occupation	Work	28 (15.1)	24 (12.9)	0.266	26 (14.0)	26 (14.0)	0.312	27 (14.5)	25 (13.4)	0.069
	Study and work	60 (32.3)	74 (39.8)		56 (30.1)	78 (41.9)		50 (26.9)	84 (45.2)	

*Based on Pearson's chi-square, p < 0.05 (significant association).

Regarding dental professionals, depression was significantly associated with marital status (p = 0.014), academic level (p = 0.005), history of COVID-19 (p < 0.001) and COVID-19 symptomatology (p < 0.001). In addition, anxiety was significantly associated with age group (p = 0.038), academic level (p = 0.008), history of COVID-19 (p = 0.002), COVID-19 symptomatology (p = 0.003) and having had a close relative with COVID-19 (p = 0.005). Finally, stress was significantly associated with history of COVID-19 (p = 0.002), COVID-19 symptomatology (p = 0.014), having had a close relative with COVID-19 (p = 0.036) and occupation (p = 0.015) (Table 4).

**Table 4:** Depression, Anxiety and Stress associated with sociodemographic factors of dental professionals in a Lima population during the second wave of COVID-19.

Variable	Category	Depression			Anxiety			Stress		
		Yes	No	p	Yes	No	p	Yes	No	p
		f (%)	f (%)		f (%)	f (%)		f (%)	f (%)	
Gender	Male	25 (13.7)	76 (41.8)	0.203	30 (16.5)	71 (39.0)	0.296	27 (14.8)	74 (40.7)	0.483
	Female	27 (14.8)	54 (29.7)		30 (16.5)	51 (28.0)		18 (9.9)	63 (34.6)	
Age group	< 30 years	17 (9.3)	32 (17.6)	0.267	22 (12.1)	27 (14.8)	0.038	16 (8.8)	33 (18.1)	0.132
	≥ 30 years	35 (19.2)	98 (53.8)		38 (20.9)	95 (52.2)		29 (15.9)	104 (57.1)	
Marital status	Unmarried	36 (19.8)	64 (35.2)	0.014	39 (21.4)	61 (33.5)	0.056	33 (18.1)	67 (36.8)	0.004
	Married	16 (8.8)	66 (36.3)		21 (11.5)	61 (33.5)		12 (6.6)	70 (38.5)	
Monthly family income	< $1125	35 (19.2)	76 (41.8)	0.269	38 (20.9)	73 (40.1)	0.649	26 (14.3)	85 (46.7)	0.611
	≥ $1125	17 (9.3)	54 (29.7)		22 (12.1)	49 (26.9)		19 (10.4)	52 (28.6)	
Children	Yes	22 (12.1)	73 (40.1)	0.091	26 (14.3)	69 (37.9)	0.093	19 (10.4)	76 (41.8)	0.123
	No	30 (16.5)	57 (31.3)		34 (18.7)	53 (29.1)		26 (14.3)	61 (33.5)	
Academic level	Bachelor	29 (15.9)	40 (22.0)	0.005	31 (17.0)	38 (20.9)	0.008	22 (12.1)	47 (25.8)	0.081
	Magister	16 (8.8)	71 (39.0)		19 (10.4)	68 (37.4)		15 (8.2)	72 (39.6)	
	Doctor	7 (3.8)	19 (10.4)		10 (5.5)	16 (8.8)		8 (4.4)	18 (9.9)	
History of COVID-19	Yes	27 (14.8)	25 (13.7)	0.000	26 (14.3)	26 (14.3)	0.002	21 (11.5)	31 (17.0)	0.002
	No	25 (13.7)	105 (57.7)		34 (18.7)	96 (52.7)		24 (13.2)	106 (58.2)	
Symptoms of COVID-19	Asymptomatic	7 (3.8)	14 (7.7)	0.000	6 (3.3)	15 (8.2)	0.003	6 (3.3)	15 (8.2)	0.014
	Symptomatic	20 (11.0)	15 (8.2)		20 (11.0)	15 (8.2)		15 (8.2)	20 (11.0)	
	Does not refer	25 (13.7)	101 (55.5)		34 (18.7)	92 (50.5)		24 (13.2)	102 (56.0)	
Close relative with COVID-19	Yes	41 (22.5)	86 (47.3)	0.092	50 (27.5)	77 (42.3)	0.005	37 (20.3)	90 (49.5)	0.036
	No	11 (6.0)	44 (24.2)		10 (5.5)	45 (24.7)		8 (4.4)	47 (25.8)	
Living with vulnerable people	Yes	33 (18.1)	78 (42.9)	0.665	42 (23.1)	69 (37.9)	0.081	29 (15.9)	82 (45.1)	0.584
	No	19 (10.4)	52 (28.6)		18 (9.9)	53 (29.1)		16 (8.8)	55 (30.2)	
Occupation	Work	26 (14.3)	79 (43.4)	0.184	34 (18.7)	71 (39.0)	0.844	19 (10.4)	86 (47.3)	0.015
	Study and work	26 (14.3)	51 (28.0)		26 (14.3)	51 (28.0)		26 (14.3)	51 (28.0)	

*Based on Pearson’s chi-square, p < 0.05 (significant association).

Regarding dental students, it could be observed in the crude model of logistic regression analysis that no factor was significantly influential for anxiety (p > 0.05). However, the variables children (p = 0.004), symptoms of COVID-19 (p = 0.025) and close relative with COVID-19 (p = 0.029) were identified as significantly influential factors for depression. The variables gender (p = 0.031), marital status (p = 0.041), children (p = 0.002) and close relative with COVID-19 (p = 0.034) were also identified as significantly influential factors for stress. Therefore, we considered adjusting the model for depression and stress variables, finding that men were 56% less likely to develop stress than women (OR 0.44; CI 0.22–0.85) (p = 0.015), while those who were not married had 81% less likely to develop stress than those who were married (OR 0.19; CI 0.04–0.85) (p = 0.029). In addition, those who had children were 83% less likely to develop stress (OR 0.17; CI 0.06–0.52) (p = 0.002) and 65% less likely to develop depression (OR 0.35; CI 0.15–0.80) (p = 0.013) than those without children. Likewise, those who became asymptomatic with COVID-19 were 60% less likely to develop depression than those who did not refer having COVID-19 (OR 0.40; CI 0.17–0.92) (p = 0.032). Finally, those who had a close relative with COVID-19 were almost three times more likely to develop depression (OR 2.96; CI 1.29–6.79) (p = 0.010) and twice more likely to develop stress (OR 2.49; CI 1.07–5.78) (p = 0.035) than those without a close relative with COVID-19 (p = 0.035) (Table 5).

**Table 5:** Multivariate logistic regression model of Depression, Anxiety and Stress in dental students according to their associated factors.

Variable	Category	Crude model												Adjusted model							
		Depression				Anxiety				Stress				Depression				Stress			
		p	OR	95% CI		p	OR	95% CI		p	OR	95% CI		p	OR	95% CI		p	OR	95% CI	
				LL	UL			LL	UL			LL	UL			LL	UL			LL	UL
Gender	Male	0.154	0.61	0.31	1.20	0.042	0.49	0.25	0.97	0.031	0.47	0.23	0.93					0.015	0.44	0.22	0.85
	Female		1.00				1.00				1.00								1.00		
Age group	< 30 years	0.855	1.08	0.47	2.47	0.904	0.95	0.42	2.14	0.884	0.94	0.40	2.21								
	≥ 30 years		1.00				1.00				1.00										
Marital status	Unmarried	0.101	0.29	0.06	1.28	0.458	0.60	0.16	2.30	0.041	0.20	0.04	0.93					0.029	0.19	0.04	0.85
	Married		1.00				1.00				1.00								1.00		
Monthly family income	< $1125	0.976	1.02	0.32	3.27	0.632	0.76	0.24	2.36	0.669	0.77	0.24	2.53								
	≥ $1125		1.00				1.00				1.00										
Children	Yes	0.004	0.22	0.08	0.62	0.624	0.80	0.33	1.94	0.002	0.16	0.05	0.52	0.013	0.35	0.15	0.80	0.002	0.17	0.06	0.52
	No		1.00				1.00				1.00				1.00				1.00		
History of COVID-19	Yes	0.377	0.48	0.09	2.46	0.421	1.86	0.41	8.48	0.516	0.56	0.09	3.29								
	No		1.00				1.00				1.00										
Symptoms	Asymptomatic	0.025	0.17	0.03	0.80	0.071	0.27	0.06	1.12	0.347	0.44	0.08	2.43	0.032	0.40	0.17	0.92				
	Symptomatic	0.500	0.55	0.09	3.18	0.834	1.19	0.23	6.11	0.599	0.60	0.09	3.98	0.416	1.37	0.64	2.90				
	Does not refer		1.00				1.00				1.00				1.00						
Close relative with COVID-19	Yes	0.029	2.72	1.11	6.67	0.275	1.63	0.68	3.89	0.034	2.76	1.08	7.06	0.010	2.96	1.29	6.79	0.035	2.49	1.07	5.78
	No		1.00				1.00				1.00				1.00				1.00		
Living with vulnerable people	Yes	0.101	1.78	0.89	3.53	0.848	1.07	0.54	2.11	0.074	1.89	0.94	3.79								
	No		1.00				1.00				1.00										
Occupation	Study	0.248	0.65	0.31	1.35	0.152	0.59	0.29	1.22	0.125	0.56	0.26	1.18								
	Study and work		1.00				1.00				1.00										

*p < 0.05significant association according to regression model (a: was not significant in the adjusted model). a: Gender was not included in the adjusted model for anxiety as a p > 0.05 was obtained. The model effect size (Nagelkerke's R2) for depression was 0.210 and for stress was 0.214.

Regarding dental professionals, it could be observed in the crude model of logistic regression analysis that no factor was significantly influential for depression (p > 0.05) and anxiety (p > 0.05). However, marital status (p = 0.019) and occupation (p = 0.023) were identified as significantly influential factors for stress. Therefore, we considered adjusting the model for the stress variable taking into account the variables marital status and occupation, finding that the unmarried were almost three times more likely to develop stress than married ones (OR 2.93; CI 1.38–6.23) (p = 0.005). In addition, those who only worked were twice more likely to develop stress than those who worked and studied (OR 2.37; CI 1.17–4.78) (p = 0.016). (Table 6).

**Table 6:** Multivariate logistic regression model of Depression, Anxiety and Stress in dental professionals according to their associated factors.

Variable	Category	Crude model												Adjusted model			
		Depression				Anxiety				Stress				Stress			
		*p	OR	95% CI		*p	OR	95% CI		*p	OR	95% CI		*p	OR	95% CI	
				LL	UL			LL	UL			LL	UL			LL	UL
Gender	Male	0.574	0.81	0.38	1.71	0.542	0.80	0.39	1.64	0.459	1.35	0.61	3.02				
	Female		1.00				1.00				1.00						
Age group	< 30 years	0.188	0.54	0.22	1.35	0.920	0.96	0.41	2.23	0.554	0.76	0.31	1.88				
	≥ 30 years		1.00				1.00				1.00						
Marital status	Unmarried	0.409	1.53	0.56	4.16	0.780	1.15	0.44	2.97	0.019	3.73	1.24	11.19	0.005	2.93	1.38	6.23
	Married		1.00				1.00				1.00				1.00		
Monthly family income	< $1125	0.989	0.99	0.43	2.30	0.518	0.77	0.35	1.69	0.177	0.55	0.23	1.31				
	≥ $1125		1.00				1.00				1.00						
Children	Yes	0.772	0.87	0.35	2.17	0.541	0.76	0.32	1.81	0.625	1.27	0.49	3.26				
	No		1.00				1.00				1.00						
Academic level	Bachelor	0.197	0.47	0.15	1.48	0.879	0.92	0.32	2.62	0.757	1.20	0.38	3.77				
	Magister	0.428	1.58	0.51	4.93	0.095	2.41	0.86	6.74	0.223	2.00	0.66	6.13				
	Doctor		1.00				1.00				1.00						
History of COVID-19	Yes	0.177	5.73	0.45	72.37	0.282	3.86	0.33	45.16	0.273	4.08	0.33	50.49				
	No		1.00				1.00				1.00						
Symptoms of COVID-19	Asymptomatic	0.358	3.14	0.27	35.95	0.282	3.65	0.35	38.51	0.430	2.63	0.24	28.99				
	Symptomatic	0.997	1.01	0.07	13.58	0.853	1.27	0.10	16.03	0.790	1.42	0.11	19.28				
	Does not refer		1.00				1.00				1.00						
Close relative with COVID-19	Yes	0.699	1.20	0.48	2.98	0.114	2.01	0.84	4.80	0.128	2.15	0.80	5.75				
	No		1.00				1.00				1.00						
Living with vulnerable people	Yes	0.985	1.01	0.47	2.15	0.131	1.75	0.85	3.60	0.577	1.25	0.57	2.77				
	No		1.00				1.00				1.00						
Occupation	Work	0.214	1.60	0.76	3.33	0.925	1.03	0.51	2.08	0.023	2.43	1.13	5.22	0.016	2.37	1.17	4.78
	Study and work		1.00				1.00				1.00				1.00		

*p < 0.05, significant association according to regression model. The model effect size (Nagelkerke's R2) for stress was 0.225.

According to the binary logistic regression analysis, it was possible to build two predictive models for depression development (β0 [coefficient of determination] = 0.821 [constant]) and stress (β0 = 5. 662 [constant]) in dental students, with the predictive variables for depression being: having children (β1 = − 1.054, X1 = 1 [Yes]), COVID-19 symptoms (β2 = − 0.922, X2 = 1 [Asymptomatic]) and close relative with COVID-19 (β3 = 1.085, X3 = 1 [Yes]). While the predictor variables for stress were: gender (β1 = − 0.826, X1 = 1 [Male]), marital status (β2 = − 1.674, X2 = 1 [Unmarried]), having children (β3 = − 1.767, X3 = 1 [Yes]) and close relative with COVID-19 (β4 = 0.910, X4 = 1 [Yes]). Regarding dental professionals, a predictive model for stress development could be constructed (β = − 0.829 [constant]), with the predictor variables being marital status (β1 = 1.076, X1 = 1 [Unmarried]) and occupation (β2 = 0.861, X2 = 1 [Only works]) (Table 7).

**Table 7:** Building predictive models for depression, anxiety and stress.

Predictive model	Variable to predict (Y*)
$\frac{1}{{1 + e^{{ - f\left( {\beta_{0} + \beta_{1} x_{1} + \beta_{2} x_{2} + \cdots \beta_{n} x_{n} } \right)}} }}$	Y
$\frac{1}{1+{e}^{-[0.821 - 1.054\,\, \left(with \,\,children\right) - 0.922 \,\,\left(COVID-19 \,\,asymptomatic\right) + 1.085 \,\,\left(having \,\,family \,\,member \,\,with \,\,COVID-19\right)]}}$	Depression in students
$\frac{1}{{1 + e^{{ - [5.662 - 0.826\,\,\left( {male} \right) - 1.674\,\,\left( {unmarried} \right) - 1.767\,\,\left( {with\,\,children} \right) + 0.910\,\,\left( {having\;family\;member\;with\;COVID - 19} \right)]}} }}$	Stress in students
$\frac{1}{1+{e}^{-[-0.829 + 1.076\,\, \left(unmarried\right)+ 0.861\,\, \left(only works\right)]}}$	Stress in professionals

Y*: dependent variable (depression or stress), e: base of natural logarithm, f_(x)_: function of probable cause (x = predictor variable), β_0_: constant coefficient of determination, Β_n_: coefficient of determination of independent variable. Note: The categories of predictor variables included in the model should take value 1; any other category of variable X that was not considered as predictor, should be considered with valor 0.

## Discussion

The Occupational Safety and Health Administration (OSHA) has placed dental professionals and students in the "very high risk of exposure" category because they are exposed to known or suspected sources of SARS-Cov-2 virus during specific procedures that generate contaminated bioaerosols^17–19^. For this reason, the performance of clinical procedures produces significant physical and psychological stress on dental students and/or dental care professionals, leading to burnout, depression, stress and anxiety^12^. As a result, this psychological impact could weaken their immunity and make them more prone to health problems^11,34–36^, and could have repercussions on their academic and professional performance^12^. For the above reasons, the present study aimed to assess how the COVID-19 pandemic affected dental professionals and students in relation to the level of anxiety, stress and depression.

When comparing the levels of depression, anxiety and stress between dental students and dental professionals, the students presented significantly higher levels of these disorders, perhaps because during the pandemic, the students had a lack of interpersonal communication due to social distancing^11^. In addition, they had to deal with online learning, having to adapt to technology and face problems such as not having a stable Internet connection^11^. Likewise, if we consider that virtual education has certain limitations in clinical training, which mainly requires manual practice^37^, uncertainty may have been generated in them regarding the development of their skills with patients^13^. This is in agreement with what was reported by Ali et al. who concluded that the students were the ones who experienced the greatest stress^11^. Likewise, our findings agree with Odriozola et al. who reported significantly higher levels of stress, anxiety and depression in students compared to the different groups of university employees^13^. Furthermore, it was observed in the present study that 41.4%, 44.1% and 47.3% of dental students presented stress, anxiety and depression, respectively; these results were slightly higher than those obtained by Hakami et al. who found a prevalence of stress and anxiety of 34.9% and 37.0%, respectively^12^. Similarly, the results obtained in the present study were slightly higher than those reported by Santabarbara et al. who in two meta-analyses of research conducted on dental students in relation to the prevalence of anxiety and depression, found prevalence of 35% and 37%, respectively^24,25^. On the other hand, in the present study dental students reported 44.1% and 47.3% for anxiety and depression respectively. When comparing these values with results obtained in the meta-analysis carried out by Batra et al. in health professionals, it was found that anxiety in nurses was 39.3% and in physicians 32.5%, obtaining 42.4% and 39.1% for depression in the same professionals, respectively^38^. The higher percentages we obtained could be explained by the fact that university students are more susceptible to psychological disorders due to several challenges they are exposed to^8^, which are further increased by the clinical procedures inherent to dental profession.

In the present study, regarding dental students, it was observed that according to gender, males were less likely to develop stress than females, which is similar to the findings of Hakami et al.^12^ and Ali et al.^11^ who found that female students were more depressed, anxious and stressed than males. This could be explained by the studies of Farooq et al.^39^ and Holtzman et al.^40^, who reported that females were more sensitive to particular situations under pressure. Another reason according to Hernández et al.^41^ could be neuroticism (trait of being anxious and emotionally vulnerable) which is more common in women. However, these findings differ from those reported by Cao et al.^14^ and Cayo et al.^10^ who reported no significant difference according to gender, probably due to the fact that both studies were conducted before mid-2020, when the infodemic was widely disseminated in social networks and broadcast media^42^, which could have generated a similar psychological impact on both genders when faced with a totally new situation. Regarding marital status, it was found that the unmarried were less likely to develop stress than the married, which could be due to the fact that students who have partners or spouses have the complicated role of maintaining their relationship and sharing time between their families and their academic studies^12^. Those who had children were less likely to develop stress and depression than those who did not have children. These findings are justified since students living alone experience higher levels of depression, anxiety and stress compared to those who live with 2 or more people^12^. In addition, having their children at home under constant supervision may have influenced them to have a certain degree of peace of mind. On the other hand, those who became ill with COVID-19 asymptomatically were less likely to develop depression than those who did not report becoming ill with COVID-19. This could be due to the fact that the first group had previous experience and acquired immunity to the disease, giving them a sense of security and confidence, in contrast to those who had not yet become ill. Finally, those who had close relatives with COVID-19 were almost three times more likely to develop depression and twice as likely to develop stress than those who did not have a close relative with COVID-19. This is consistent with that reported by Cao et al.^14^ who found that college students' anxiety about the pandemic was significantly associated with the presence of a family member or acquaintance infected with COVID-19.

With regard to the dental professionals in this study, 24.7%, 33.0% and 28.6% presented stress, anxiety and depression, respectively; these findings are discordant with those indicated in the study by Nayak et al. in which they refer values of 17.97%, 56.2% and 42.28%, respectively. This is probably due to the fact that this study was carried out between May and June 2020^43^, a time of greater uncertainty since vaccines were not yet available and social interaction was restricted, which could have generated higher levels of anxiety and depression^23^. For 2021, the lower prevalence percentages we found could be due to the positive adaptation of professionals to a context of adversity (resilience) as reported by Barzilay et al.^44^, Parvar et al.^45^ and Cayo et al.^46^. In reference to the factors "children" and "close relative with COVID-19", our findings agree with those obtained by Li et al.^47^ who reported that among the risk factors for developing stress, anxiety and depression is the number of children, with those who had two or more children being more susceptible. In addition, they also reported that having family members or relatives with suspected or confirmed COVID-19 was one of the risk factors for depression and stress. Regarding age, our findings agree with Owen et al.^21^ who indicated lower levels of stress in the 18–24 age group compared to other age groups. However, they differ with Nayak et al.^43^ who reported that those aged 30 years and younger had higher levels compared to the other age groups. This is probably because Nayak et al.^43^ conducted their study between May and June 2020 unlike Owen et al.^21^ and the present study which were conducted in 2021. The timing of the survey may have influenced the lack of resilience of younger people in this complex circumstance^48^ and the lack of socialization^49,50^ generating a greater psychological impact. On the other hand, the older the age, the greater the responsibilities and the greater the job and economic prospects, which could lead to increased worries and uncertainty. With regard to marital status, unmarried dental professionals were almost three times more likely to develop stress than married ones, which coincides with Nayak et al.^43^ who found a significant association between stress and marital status, where single people had higher scores than married ones. This may be explained by the fact that loneliness is a risk factor for developing mental disorders under situations of constant pressure^51^, and added to this, the social isolation resulting from the pandemic^2,49,50^, may have induced them to demand greater adaptation than usual (stress). In reference to occupation, it was found that those who only worked were twice as likely to develop stress as those who worked and studied. This result can be explained by the fact that they were exposed to greater psychological distress due to the type of care provided by dentists^19^, dealing with different people who have different demands and expectations^52^, educed income due to the lower number of visits^5^, and reduced work time due to the fear of becoming infected. On the other hand, those who worked and studied shared their time in both activities, reducing the period of clinical work and therefore had less risk of exposure to the virus, which could have given them a greater sense of peace of mind.

It has been reported that prolonged and untreated psychological disorders would result in high levels of burnout in the future^11,21,34,35^. This study is important because some sociodemographic factors have been found to predict the development of depression in dental students and stress in dental students and professionals using two predictive models in students and one predictive model in Peruvian dental professionals, after they faced the highest COVID-19 fatality rate worldwide during the second wave of infection^16^. Furthermore, in the present study, unlike other studies, it was observed that the type of dedication of the professionals was an influential factor significantly related to the development of stress. Likewise, having been asymptomatic when they became ill with COVID-19 was a protective factor for depression in the students. These results may be useful for competent authorities to promote strategies to provide adequate psychological support, which is vital for students and health professionals, since providing mental health support is as important as physical protection during the pandemic.

The present study had some limitations, such as not being able to evaluate the respondents in person, since at the time the survey was conducted, the country was in a national emergency and social isolation was mandatory. Likewise, students from all academic years were not taken into account because first year students take basic science subjects that could be taken virtually. In addition, since they are not required to attend patients and perform dental procedures, they might not be exposed to higher levels of depression, stress and anxiety since they do not need to develop manual care skills^37^ and are not at risk of exposure to biocontaminated aerosols. On the other hand, it should be recognized that due to the sample size used in this study, the predictive models generated should be taken with caution, since it is necessary to assess in future studies with population-based design other possible predictor variables according to social, political and economic realities in which dental students and professionals develop.

It is recommended that the levels of depression, anxiety and stress in dental professionals and students in different geographical areas be compared to improve knowledge in this regard, since Cao et al.^14^ suggest that living in urban areas is conducive to reducing anxiety. On the other hand, longitudinal studies are needed to evaluate the psychological impact of COVID-19 and the level of acceptance of the therapeutic support received. In this regard, it is suggested that the authorities of professional schools and universities take into account the organization of timely plans and strategies for mental health care and physical protection in the pandemic context^20^.

## Conclusions

In summary, with the limitations of this cross-sectional study, it can be concluded that dental students from two universities in the Peruvian capital had a higher prevalence of depression, anxiety and stress during the second wave of COVID-19; in addition, having children and being asymptomatic were protective predictors for depression, while being male, unmarried and with children were protective predictors for stress. However, having had a close relative with COVID-19 was a risk predictor for depression and stress. In dental professionals, only working and being unmarried were risk predictors for stress. The higher frequency of psychological disorders obtained for students indicates the greater impact that COVID-19 pandemic had on them. It is important that psychological support and intervention programs that promote resilience are managed in a timely manner for this population in order to facilitate post-traumatic growth. On the other hand, none of the factors considered in this study was a predictor for anxiety in both dental students and dental professionals.

## Data Availability

All data analyzed during this study are available from the corresponding author on reasonable request.

## Abbreviations

COVID-19: Coronavirus disease 2019
CI: Confidence interval
DASS: Depression, Anxiety and Stress Scale
OR: Odds ratio
OSHA: Occupational Safety and Health Administration
STROBE: Strengthening the Reporting of OBservational studies in Epidemiology
SPSS: Statistical Package for the Social Sciences
